# UNDERSTANDING AGE DIFFERENCES IN GRIEF SUPPORT NEEDS: RESULTS FROM A COMMUNITY-WIDE SURVEY

**DOI:** 10.1093/geroni/igad104.1784

**Published:** 2023-12-21

**Authors:** Caroline Collins-Pisano

**Affiliations:** University of Colorado Colorado Springs, Colorado Springs, Colorado, United States

## Abstract

Experiences of grief and bereavement differ across the lifespan. As such, needs for community grief services may vary based on age. This presentation outlines age differences found in the community-wide survey conducted during the Exploration and Preparation phases of the EPIS framework. A total of 668 community members within an age range of 18-65+ completed the survey. The majority of participants were 18-29 (n = 358, 54%), 28% were 65 or older (n = 186), 11% ages 50-64 (n = 71), and 8% ages 30-49 (n = 52). Survey results revealed both age differences and similarities in experiences of loss, participation in past grief services, likelihood of participating in future grief services, and barriers to participating in services. Respondents aged 65 or older experienced significantly greater lifetime loss compared to other age groups, with 100% reporting having experienced the death of someone significant to them. Yet, older adults and adults aged 30-64 reported similar rates of participation in past grief services. However, while older adults shared the least barriers to participating in grief services, they reported similar likelihoods of participating in a variety of future grief services as adults aged 30-49 or 50-64. The survey demonstrated that while older adults experienced the greatest amount of loss, they did not report the highest overall likelihood of utilizing grief services. Rather, each age-group presented a distinct and nuanced hierarchy of preferred grief services and needs, the details of which will be explained further in this presentation.

